# The Primary Reasons People Participate in Maternal‐Fetal Phase I Clinical Trials Are Psychosocial

**DOI:** 10.1002/eahr.70025

**Published:** 2026-11-06

**Authors:** Julia E. H. Brown, Leslie S. Riddle, Emma Canepa, Billie Lianoglou, Dawn Gano, Teresa N. Sparks, Jodi Halpern

**Affiliations:** ^1^ Senior research fellow at the Monash Bioethics Center at Monash University; ^2^ Researcher at the University of California, San Francisco; ^3^ Program manager for the Center of Genome Surgery at the University of California, San Francisco; ^4^ Perinatal genetic counselor at the University of California, San Francisco; ^5^ Division head of pediatric neurology and a professor in the Department of Pediatrics at the University of British Columbia and BC Children's Hospital; ^6^ Associate professor of maternal‐fetal medicine and clinical genetics at the University of California, San Francisco; ^7^ University Chair, Class of 1940 Endowment, and a professor of bioethics and medical humanities at the University of California, Berkeley

**Keywords:** human subjects research, human research ethics, pregnancy and research, in utero research, rare genetic conditions, in utero somatic cell gene therapies

## Abstract

Recent critiques of ethical guidance for maternal‐fetal therapy clinical trials has highlighted the need to weigh maternal, fetal, and societal interests, respectively, while deprioritizing psychosocial reasons for participation. Psychosocial reasons refer to the psychological and social influences on study participants’ decisions, and they are often central to considerations about informed consent. However, psychosocial reasons are described as too indirect, subjective, and vague to incorporate into prenatal clinical and regulatory frameworks. This qualitative study examined the perspectives of 11 individuals (within eight couples) enrolled in one of two maternal‐fetal therapy phase I clinical trials for rare genetic conditions. Interviews revealed that motivations to participate were primarily psychosocial, including existential concerns given prior bereavement and/or fear of loss; temporal control through taking action as opposed to feeling passive; hope and optimism; and intergenerational and social motivations given one's family experience with disease and desire for social connections. Psychosocial reasons for participating ought to be given more consideration in ethical frameworks for maternal‐fetal therapy phase I clinical trials, as well as in the approach to informed consent. It is important for clinical research teams to be especially conscientious to ensure appreciation for participants’ reasons for participating that extend beyond typical frameworks of clinical risk/benefit, and to support participants who might harbor optimistic expectations of medical procedures as they transition to postnatal realities.

Experimental in utero treatments have been conducted for the past half‐century under varied regulatory and ethical guidance for clinical‐research teams. Maternal‐fetal medical interventions targeting the fetus aim to improve, if not prevent, disease outcomes for the future child. Experimental in utero treatments advance scientific knowledge with the goal of establishing fetal interventions that give pregnant women and their families more options when faced with fetal diagnoses that require attention during a pregnancy. Certain types of in utero surgical repairs and blood transfusions are now a standard of care, with clinical trials of in utero stem cell transplants and enzyme/protein replacement therapies paving the way toward in utero somatic cell (i.e., in vivo, nonheritable) gene therapies. Considering prior trials of experimental maternal‐fetal interventions with uncertain benefit‐to‐risk ratios, there are many ethical lessons learned from analysis of cost‐benefit ratios and the views of pregnant women and their families regarding treatment outcomes.^1^ In the interests of future investigators and prospective participants, it is critical to account for whether the lived experiences of pregnant women and their families resonate with or challenge current ethical guidelines and relevant regulations when an experimental in utero treatment is being conducted as part of a clinical trial.

Regulations in the United States governing research with pregnant women, fetuses, and neonates may be inadequate to address the complexity of factors that impact decision‐making about whether to participate in maternal‐fetal therapy trials. Consistent with the approach generally used for all types of clinical trials testing therapeutic interventions, the federal regulations focus solely on objective maternal‐fetal safety and efficacy measures. The regulations permit pregnant women to participate in phase I trials when preclinical data suggest potential benefit to the future child or, in the absence of direct benefit to the future child, there is no more than minimal risk to the fetus and pregnant person and there is the prospect of gaining relevant scientific knowledge.^2^ However, unaccounted for in these regulations are many ethical and social concerns, ranging from the inseparability of fetal and maternal interests to how to account for issues concerning equity, reproductive rights, and psychosocial factors.^3^, ^4^, ^5^ Historically, maternal‐fetal surgical innovations did not prioritize the pregnant woman; the focus was primarily on the fetus as the patient rather than the pregnant woman.^6^ In the context of maternal‐fetal trials, excluding indirect maternal, fetal, and societal risks and benefits from regulatory calculus is an oversight.^7^ As we move toward the era of prenatal somatic genome editing, it is increasingly important for researchers to engage relevant patient communities to understand what notions of benefit mean to them.^8^

Benefit‐to‐risk ratios may not capture the interrelated psychosocial reasons why families decide to participate in maternal‐fetal clinical trials. Psychosocial factors refer to the psychological and social influences and impacts on decisions to participate. Previous research has suggested that psychosocial factors are “indirect,”^5^ with individual motivations and values rendered too subjective and secondary to questions of safety.^9^ Parental agency and relief from having a third option aside from postnatal treatment or pregnancy termination has been considered too “limited in magnitude and duration” to be accounted for in ethics frameworks.^10^ Empirical evidence of lived experiences based on contemporary medicine and clinical trials is necessary to better define and include psychosocial benefits and risks in ethical and medical frameworks.^10^ This would also improve participant counseling, as information could be more personalized to the values and experiences of families.

Obtaining participant perspectives about phase I maternal‐fetal therapy clinical trials can identify primary reasons for participating and whether these reasons overlap with regulatory priorities and resultant ethical frameworks of accepting risks in the setting of unknown benefit. Uncovering the primary reasons for participating in phase I trials means tracing back to the origins of understanding and belief systems that inform those reasons and ultimately support informed consent to participate.^11^ Our qualitative study examined the motivations

**This qualitative study demonstrates that psychosocial reasons are central for families enrolling in maternal‐fetal phase I clinical trials.**

and experiences of 11 individuals, within eight couples, who enrolled in phase I maternal‐fetal therapy clinical trials. We discuss how psychosocial reasons for trial participation were central to the women/families, and how benefit‐to‐risk language in clinical terms may be limited in applicability to the decision‐makers (participants) themselves.

## RESEARCH CONTEXT AND STUDY METHODS

Participant families were enrolled in one of two phase 1 clinical trials during the second to third trimesters of pregnancy: in utero hematopoietic stem cell transplant (IUHCT) for alpha thalassemia major (ATM) or in utero enzyme replacement therapy (IUERT) for lysosomal storage diseases (LSDs). Individuals with ATM are unable to produce functional hemoglobin, resulting in severe anemia in utero. As a result, ATM can result in hydrops fetalis, which is a condition of abnormal fluid overload in the fetus that carries high risks of fetal demise and maternal morbidity.^12^ Many fetuses with ATM will not survive to birth or will die shortly after birth without in utero blood transfusions (IUTs) to treat the underlying anemia.^13^ After birth, children with ATM may undergo hematopoietic stem cell transplantation as a cure for this condition. Variants leading to ATM are especially common in Southeast Asia, including Southern China, Vietnam, and Thailand, where up to 40% of the population are carriers for the condition.^14^ Based on preclinical models suggesting that IUHCT using human maternal stem cells could be used to allow for engraftment without conditioning and lead to durable tolerance to transplanted cells postnatally,^15^ a phase I trial for IUHCT was initiated. While safe and well tolerated, enrollment ended early due to interim data suggesting a lack of efficacy of the IUHCT.^16^ Notably, this trial allowed for a companion ATM patient registry and enabled the collection of clinical data from patients electing for IUTs management due to a prenatal diagnosis of ATM. These data demonstrated benefits of IUTs including resolution of hydrops, later‐term delivery, and neurodevelopmental outcomes comparable to peers.^13^ This evidence supported the development of new clinical guidelines which included the option of fetal management with IUTs for ATM where historically patients were informed the diagnosis was lethal and counseling was limited to pregnancy termination or awaiting perinatal demise.^17^

The second phase I clinical trial is ongoing and is studying the safety, efficacy, and clinical impact of IUERT for LSDs. Through this trial, enzyme replacement is delivered through the umbilical vein to fetuses known to have one of eight LSDs with a US Food and Drug Administration‐approved intravenous enzyme replacement available postnatally. These conditions are all monogenic multisystem disorders characterized by progressive cellular damage, organ and central nervous system dysfunction, and severe clinical manifestations.^18^ Fetuses with some of these LSDs may also be at increased risk of developing hydrops fetalis along with maternal complications such as mirror syndrome.^19^ These conditions are considered pan‐ethnic, with some disease‐causing variants that are better studied and known to be enriched in specific populations, and other genetic variants leading to LSDs that are understudied across populations.^20^ Based on preclinical data suggesting that IUERT may address limitations of standard of care postnatal ERT such as the inability to cross the blood‐brain barrier,^21^ this (ongoing) clinical trial seeks to understand how IUERT may mitigate irreversible, prenatal damage to developing organs.

Both clinical trials involve extensive and ongoing discussions among our multidisciplinary research team (including JB, EC, BL, DG, TS), with many additional subspecialties, such as maternal‐fetal medicine, clinical genetics, and bioethics, represented. Over the course of multiple meetings with families before they provided informed consent to participate in a clinical trial, members of our team who participated in obtaining informed consent (including EC, BL) reviewed with the families the primary study aims of safety and efficacy, while recognizing that the interventions under study were not expected to result in a cure. Therefore, participants were asked to undergo the study‐related prenatal interventions in addition to pursuing standard of care treatments after delivery, which include serial transfusions for ATM or serial administration of ERT for children with LSDs. Other disease‐modifying therapies after birth may also be considered, such as bone marrow transplant (BMT) for ATM or innovative clinical trials including gene therapy for children with mucopolysaccharidoses (MPS). During the informed consent process, multidisciplinary pediatric experts provided education to the families about the disease process and reviewed expectations for postnatal outcomes and ongoing therapies.

All potential participants received nondirective counseling about their options: pregnancy termination, continuing the pregnancy without intervention, or continuing the pregnancy and enrolling in the clinical trial. Access to termination of pregnancy is an important part of both clinical care and in utero clinical trial participation, for reasons of reproductive autonomy, clinical safety, and scientific progress.^22^ How willingness to consider termination influences clinical trial participation and feelings about postnatal treatment is beyond the scope of this paper, but as we note in table 1, there was varied willingness to consider termination amongst those we interviewed. Since all participant pregnancies included in this study resulted in live births, participant responses must be viewed within the context of live births.

**Table 1:** Participant Demographics, Clinical Situation, and Interview Context

Pseudonym(s)	Nationality and self‐identified race/ ancestry	Health insurance status	Disease history	Relevant pregnancy history	Clinical trial	Willing to consider termination	Time of interview
Sasha & Nathan	American, Black African American (both)	US Medicaid	Fetus with MPS II (Hunter); brother (living); great uncles (deceased)	1 prior miscarriage	IUERT	Yes	35 weeks gestation
Hamid & Mariyam	Pakistani, South Asian (both)	Private	Fetus in most recent pregnancy with MPS I (Hurler); first‐born (died at age 7 of post‐BMT complications)	2 of 4 pregnancies affected by MPS I	IUERT	Yes, if abnormality diagnosed earlier	3 months after delivery
Juanita	American, Southeast Asian	Private	Fetus with ATM	None	IUHCT	Yes	4 years after delivery
Lisa	American, Southeast Asian	Private	Fetus with ATM	1 prior unaffected pregnancy	IUHCT	Yes	18 months after delivery
Claudia	American, Native Hawaiian	Private	Fetus with ATM	1 prior unaffected pregnancy	IUHCT	No	5 years after delivery
Penny	Canadian, Southeast Asian	Canadian government	Fetus with ATM	None	IUHCT	No	17 months after delivery
Madelaine	American, Southeast Asian	Private	Fetus with ATM	1 prior neonatal loss at 35 weeks due to ATM associated complications; 1 subsequent pregnancy, unaffected with carrier status	IUHCT	No	2 years after delivery
Hao & Rose	Canadian, Southeast Asian (both)	Canadian government	Fetus with ATM	1 miscarriage; 1 termination of pregnancy with fetal ATM	IUHCT	Yes	9 weeks after delivery

From those enrolled in the clinical trials, 11 individuals across eight couples (eight maternal participants, three reproductive partners) were interviewed separately for 90 minutes (by JB) after the conclusion of the fetal interventions. These participants were made up of all families who had enrolled in the IUHCT trial, and two families enrolled in the IUERT trial who were available for and willing to be interviewed at the time of JB's fieldwork. Coauthor clinical team members who participated in the informed consent process for the clinical study (EC, BL) assisted in recruitment but did not participate in the consent process for the interviews, nor the interviews themselves and subsequent coding. Thematic coding and analysis were conducted by JB and LR to determine key drivers of decision‐making and participants’ conceptions of benefits and risks. Coauthor clinical team members (EC, BL, DG, TS) situated the analysis within knowledge of the clinical trials, related clinical care, and the maternal‐fetal medicine field. The timing of the interviews, up to five years postpartum in one case, allowed us to evaluate the potential for decision regret and therapeutic misconception or misunderstanding. Additionally, as most participants were part of the IUHCT trial (n = 7; 63%) and interviewed after the trial was discontinued, this allowed exploration into how families felt about the outcome of the trial. The cohort was geographically and demographically diverse across North America (see table 1), although the majority of those enrolled in the IUHCT trial were of Asian ancestry. Pseudonyms were used for parents, and also fetuses and children when mentioned by name.

## FINDINGS AND DISCUSSION

Interviewees suggested that clinical trial participation decisions involved psychosocial reasoning that did not neatly map onto clinical or scientific reasoning. Decision‐making at the time, as described below, was made while primarily focused on immediate needs. We identified four key subthemes related to motivations for participation: (1) existential fear given prior bereavement and/or fear of fetal loss; (2) temporal control through taking action; (3) hope and optimism; and (4) intergenerational and social connections given family experience with disease and desire for social connections.

### “Nothing could be worse than what I had to go through”: Existential fear

The prospect of bereavement resulting from the loss of a fetus or newborn was an overwhelming risk of not participating, beyond consideration for clinical risks or longer‐term postpartum logistics. Most interviewees (63%) had experienced prior loss of children or fetuses from the genetic conditions targeted by the clinical trials; the decision to enroll was often made in the shadow of past loss. For others, the stakes were still high in terms of the possibility of pregnancy loss. In the neonatal intensive care context, experiences of anticipatory grief and grief associated with actual loss have long been recognized as overlapping insofar as knowing there could or will be death.^23^ In the prenatal context, similar experiences of existing and/or anticipatory grief were compounded in situations where perinatal or child death had already been experienced.

The decision to participate was viewed as an alternative to the inevitable loss and grief otherwise. Sasha and Nathan recalled that enrolling their son, Tobias, in the IUERT trial for X‐linked MPS II (Hunter syndrome) was an opportunity to counter the possibility of further grief they had endured when they lost their first pregnancy with twins. Although it was not clear if their first pregnancy had been affected by MPS II, Sasha said, “if I didn't have a miscarriage, I possibly would've terminated [the currently affected pregnancy]. But having gone through that, I couldn't allow somebody to do that willingly.” Both of Sasha's brothers also had Hunter syndrome, one of whom had died at birth, and three affected great uncles had died in childhood. Nathan, who had seen a photograph of Sasha's mother holding her baby until he passed away, recounted, “the decision was really just to get the treatment or hold him early, or don't get the treatment and just let him be delayed…. We were really considering that last option, but we decided to do the trial and at least give him a fighting chance.” Similarly, Madelaine, who had suffered the loss of her son, Thomas, at 35 weeks gestation due to hydrops fetalis associated with ATM, described two years after enrolling in the IUHCT trial: “all my choices just root back to what I went through with Thomas. I think had that not happened … I'd probably choose the same route, but … nothing could be worse than what I had to go through. So, whatever I had to endure for the trial and when, [whatever] the payout would be, [would] just outweigh anything that I would have to go through [if not taking part].”

Prior loss can shape risk tolerance. When Hamid and Mariyam had enrolled Mariyam and the fetus she was carrying in the IUERT trial targeting MPS I (Hurler syndrome), they were already parents to three children, the oldest of whom was Laila. Laila had been affected by the condition and died at age seven from medical complications following BMT pursued in Pakistan in an attempt to treat her MPS I. “Whenever we thought about [the] worst case, I mean, [it was] my daughter, Laila,” Hamid explained. Hamid and Mariyam's profound grief had become the main potential risk, associated with *not* participating in the prenatal clinical trial. Having had lived experience with Laila, “there was no other option … nobody need[ed] to tell us because we had been through this.” Regarding risks associated with the clinical trial, Hamid verified, “I'm unable to see those risks” relative to the risk of losing another child to the condition. In terms of the worst risk for them personally, “it's very easy to say, but very difficult to digest [the] death of a child, but that's so certain in [the] case of the progression of the disease.” Hamid was “very quick to analyze that option, what could be the ultimate thing going wrong? Ultimately it could be Laila, I was mentally prepared for that. Though I didn't want to feel through it.” IUHCT participant Lisa, whose fetus had ATM, echoed simply, “the alternative is that the baby isn't here.”

For bereaved and nonbereaved couples alike, participating meant trying to save a life as opposed to not trying. Lisa said, “parents and especially maybe moms are more willing to do anything to save their child…. I was going to do whatever it takes.” She elaborated, “my mindset was like, I can say that at least I tried if it didn't work out; I wanted to make sure I gave it my best effort.” Madelaine described that it was a “no‐brainer to just do the trial because I was going to fight for this baby.” Similarly, Juanita described that participating in the IUHCT trial “was a no‐brainer for us at that point, because we knew that we would at least try to do anything,” with “no reservation in terms of doing anything we can to save my child.”

The clinical outcome of participation was less of a concern for families than what it meant to try. Madeleine said that while getting a “good outcome” would be “ultimate,” “we also thought if we didn't, we could say we did everything that we could have done…. Why would you do nothing when you could do a small something?” Madelaine suggested that this desire for more agency also surpassed financial considerations if, beyond a clinical trial context they were not able to financially access a potential treatment: “I think I honestly would've done it anyway, even if I couldn't [afford it], I would figure it out, I would just figure it out, I don't know how I would do it, but my mindset was, I'm going to have this baby and I'm going to do whatever I can.”

While imagining that the long‐term commitment of weekly postnatal infusions would be “very hectic” and comparatively time consuming, taking six hours instead of one hour in utero, Hamid described the prospect of ongoing enzyme infusions as “not terrible” relative to a childhood BMT. Hamid saw BMT as “very terrible” and associated with “the worst memories of a BMT center from the third world, because most of [the children] are dying anyway.” He had previously felt let down, “very upset and angry” about information he had received elsewhere before agreeing to Laila's BMT. His perception was that a doctor told them the procedure had a 75‐85% survival rate but later learned this applied to only 20‐30 days from discharge. He saw the IUERT in sharp contrast because it was transparently a phase I clinical trial. Fearing the loss of his son, who was an IUERT study participant, during the prenatal period or shortly afterwards, Hamid sought emotional protection by trying “not to get attached to him.” Until they could reduce uncertainty by committing only to the postnatal transfusions rather than opting for another postnatal stem cell transplant, which was ultimately experimental, Hamid “didn't develop any bond with him. Because until then I thought it's very risky, it can go wrong at any point of time.”

Families tended to view discomfort and risk associated with the clinical trials in the broader context of life, for which lack of control may still be felt. IUHCT participant Penny said, “there's no negative side effects or outcomes from participating…. Okay, I got pain for a month from standing or walking because of my hip bone [from the maternal stem cell donation], but there's nothing else that's negative out of it.” She described that although she had initially felt “disappointment” upon learning there was no efficacy seen in her child, “the disappointment doesn't overwhelm us because we understand that every aspect of our lives [is] in the sovereign control of God.” Similarly, Hamid viewed other risks in the context of life more generally: “we don't know about our own lives, because there are so many uncertainties … everything has some degree of risk.” Moreover, the existential risk of inaction versus participation also meant there was a heightened focus on having a live birth, first and foremost.

### “I was just trying to get him to survive birth at that point”: Temporal control

While it may be desirable for patients to fully account for longer‐term considerations, participants in the present study highlighted how their concerns tended to be anchored in the immediate stakes of loss. Historically, maternal‐fetal surgical interventions have been criticized for a short‐sighted focus in the perinatal period rather than lifelong impacts.^24^ However, we observed that there was no decisional regret even when families were focused on the pregnancy and more immediate outcomes. Challenges in imagining the postnatal period pertained more to medical procedures that are hard to account for until they happen.

Due to immediate existential challenges, the realities of life after prenatal treatment were not at the forefront of many participants’ minds. IUHCT trial participant Rose, who had experienced two prior pregnancy losses, said that some of the inability to consider postnatal realities was due to being “scared all the time of something happening” to the pregnancy. Rose's husband, Hao, described the difficulties in adjusting afterward: “having that [distant expectation of blood transfusions] to visually see that now, the amount of pokes it takes to draw her blood, the effort it takes [to] actually just find an IV before you even start the transfusion.… I didn't imagine it would be this hard until I saw her go through it…. I didn't really consider I guess the emotional weight of that.” Hao recalled: “it never really dawned on me the time commitment that it would still take. I was prepared for it and yet it was still another level…. I didn't realize how much of an adjustment that would take.” Rose felt somewhat concerned, “are we being selfish choosing this path for her? … ‘Cause she has to suffer all of this.” Rose explained the distinction of witnessing her daughter's treatments postnatally: “It's more since she's been born, just watching her go through all of these things … when we first made the decision … I did feel like this was something that crossed my mind but … maybe it's just hitting me a lot more now seeing it…. It's so hard to watch … I didn't think it was a big deal having those in utero transfusions … I wish I could take the pokes for her, right now.”

It was challenging for families to engage in future possibilities until they were relevant. Juanita recalled: “we were constantly told about the [postnatal] transfusions every few weeks … [but] my goal was, ‘Let's make it to full term. Let's get him to breathe his first breath, and then go from there.’ … I never really thought about quality of life…. I didn't even think about insurance at the time. I was just like, “Yeah, put me on a plane. Do what you need to do.” Once the immediate decision point and threat of fetal demise was over with the in utero blood transfusions accompanying the IUHCT, Juanita recalled thinking “Okay, let's keep it going. He's doing really well. I'm doing really well, let's keep it moving…. as the trial went on, my fear or my psyche of being, ‘Oh, the worst is going to come,’ got less and less.” Penny similarly recalled how over time the procedures built a more positive experience: “[I]n the beginning … I was physically exhausted really … [but] as the routine is built and you find ways to enjoy that day rather than feel like, oh, you're just trapped in the hospital that day, it gets better.” The experience of high‐stakes health care becoming more mundane and familiar while demanding a focus on present time to prevent being psychologically overwhelmed is not unusual.^25^ With regard to the postnatal period, Juanita noted: “Psychologically, it's draining. It's so draining. And it's hard because you can't really get help with [appointments] … it's so hard to keep track of…. If it could stay in prenatal, that would be ideal, of course. But we knew going ahead of time that this would happen. What I did not know was how many different doctors he would see.” It was not until they met with a pediatric cardiologist after her son was born that Juanita became concerned about whether he would be able to run the way a toddler loves to, stating: “It's those type of things now that I think about. But during the pregnancy and during the trial, I don't think my mind even went there … I was just trying to get him to survive birth at that point.”

The question of longer‐term outcomes may be more relevant for those without prior experience of loss. Lisa, a medical professional who had one unaffected child at the time of her pregnancy and enrollment in the clinical trial, recalled of her decision: “I questioned whether even if I saved this baby, what does that mean for her if she survives?” She understood the need for postnatal transfusions for ATM regardless of the IUHCT outcome: “these kiddos develop normally, they have a good quality of life, life expectancy tends to be typical, and so there's good outcomes. It's just that chronic blood transfusions is something that we would have to deal with as well as any complications that come with that.” Penny described feeling able to “understand” postnatal needs after speaking with her hematologist, who said: “There's a lot of people with the beta thalassemia who go through transfusions all the time … you realize it's actually not as rare or daunting—people have lived their lives up to 60.”

Families in this study did not have decisional regret in the first few years following delivery. Rose, speaking nine weeks after delivery, said, “The outcome wasn't as we hoped. I still don't regret doing it because if I didn't, we'll always wonder.” Hao clarified that he and Rose's collective decision would not have been different had they known what to expect postnatally: “I think our decision … would've been the same regardless.” Madeleine, speaking two years after delivery, echoed, “At the time, I didn't know what the trial entailed…. But I wouldn't change my decision … I'd still go through it again.” Hamid, speaking three months after delivery, imagined he would tell his child one day if they questioned the decision that it was “what I deemed fit at that point of time in my utmost sincerity.”

### “I'm very optimistic to the point of unrealism”: Hope and therapeutic optimism?

Participants described being motivated by hope and optimism for outcomes that they also knew to be unlikely. It can be challenging to differentiate between hope and optimism and here we see both experienced. Hope may be understood as more of an emotion with less personal control over the outcome, whereas optimism affords action toward a goal.^26^ Participants tended to focus on the possibility of a positive personal outcome that they did not feel personal control over (hope) as the same as experiencing their participation as a personally actionable path toward the desirable outcome (optimism). It is unclear whether or not participants ultimately experienced what has been theoretically described as “therapeutic optimism”—hope (chance) and optimism (action) for benefit with full understanding (no misconception) that direct benefit was unlikely.^27^

Demonstrating both optimism and hope, Nathan felt particularly confident in the outcome of the trial because of his faith in the caring team and trustworthy institution: “I think the treatment is going to do what it's supposed to…. I one hundred percent believe in the treatment and the people at [institution], one hundred percent.” This full confidence was arguably dispositional (Nathan may be a generally optimistic person), while the contingency of the clinical team was situational (Nathan was optimistic given the supports). Situational optimism is more likely to raise concerns about informed consent because the optimism may be contingent upon the information given for the particular situation.^27^ We recognize that clinical team providers who obtain informed consent from trial participants become part of the communicative context that can undermine neutral information exchange about the trial.^28^ At the same time, caregiving as a moral experience is not adequately accounted for in bioethics principlist frameworks.^29^ Misunderstanding of the clinical trial objectives was also not evident. Nathan clarified his awareness of the fact that this was a clinical trial: “We don't know what could happen, so we're just hoping and praying that it's successful and that other families look at this and say, ‘We want to do that, give our kid a fighting chance.’” While we cannot rule out the influence of trust in the team or social relationships built through care or the power dynamic regarding the institution and future health care needs, the experience of hope and optimism was grounded in the existential and psychosocial needs of participants.

Regarding the potential for decisional regret, which could indicate insufficient comprehension of the clinical trial, it is significant that hope did not cease for the IUHCT participants even upon learning that the IUHCT had not been successful. Madeleine described of her two‐year‐old with ATM: “He's fantastic…. He is just [a] normal little boy going through the whole terrible twos stage and just hitting on the milestones.” Lisa echoed, “she's been wonderful. She has a little bit of delay. Nothing that she's not progressing at.” She continued the sentiment she felt while pregnant, “I'll do what it takes just to help her be happy and healthy.” She said of the ongoing blood transfusions, “We hoped she wouldn't need it, of course. But if that's what's going to help her, then yeah, that's not even an issue…. We're so grateful because our [nonaffected] son was a little tricky … if you take away the medical conditions, she's so much more easier and chill than him…. I can't imagine not having her here and I would do it all over again for her.” This again suggests both dispositional and situational optimism, the latter of which was propelled by the fact that Lisa's son was still alive largely thanks to her ongoing commitment to blood transfusions.

There is a clinical responsibility to hold space for what may be described as a patients’ “psychosocial and existential need” to feel hopeful, without giving false hope.^21^ If personal belief is driving hope rather than clinical information that informs this hope, it may be deemed ethical and even therapeutically important;^30^ what becomes ethically problematic are unrealistic therapeutic beliefs.^11^ In the context of fetal surgery for spina bifida, participant couples have experienced hope by imagining “the future perfect tense” where the surgery “will already have been accomplished.”^31^ Those interviewed for the present study held on to hope of an exceptional outcome until proven otherwise. Hao reflected, “There was a suspicion in the back of my mind that it probably wasn't successful … so when we found out [that it did not work], it wasn't a shock. I don't think it was even disappointing. I think we were hoping to be surprised, but … it wasn't the results that we hoped for.”

How can existential and psychosocial needs in the moment be reconciled with downstream consequences if these needs and perspectives change? At the time of the consent process for the clinical trial, holding both hope and optimism together and mitigating against unrealistic expectations is critical for both ensuring understanding and reducing the possibility of decisional regret. Informed consent for experimental maternal‐fetal therapy requires individualized counseling that respects parental desire for “precarious hope” while also presenting realistic considerations and risks.^32^ Notwithstanding concerns about a slippery slope into therapeutic misconception if greater psychosocial benefits are perceived over clinical safety concerns,^9^ we described above how a focus on the present is helpful for families and did not, within the range of this cohort's postpartum follow‐up period, lead to decisional regret. As illustrated by prior work describing experiences of prenatal exome sequencing, the temporal confinements of pregnancy with prognostic uncertainty can result in preserved hope for families.^33^ However, we are aware that this is a short time range and decisional regret needs to be studied over a longer period of time.

Once enrolled in the trial, Hamid described that he and Mariyam had “a very visible ray of hope.” The weighing of risks, benefits, and expectations of families is likely to be different before and after deciding to participate. All families had already participated in the intervention by the time they were interviewed. They were thus more likely to be in an “implementation mindset” after enrolling, rather than “deliberative mindset.”^34^ The implementation mindset is necessarily biased toward a likelihood of success. Moreover, none had experienced adverse events. Learning about the clinical trial, Hamid described a bolstered sense of hope after meeting and connecting with the clinical research team: “I'm very optimistic to the point of unrealism. I was always hoping that [we] would be a stellar example … I know it's very stupid to think like that … but … probably that's how you convince yourself. Otherwise … I won't be able to sleep at night; that's how I rationalize.”

Rose stated that her main reasoning for participating in the trial “was more the personal aspect … for sure just the chance of hope.” Claudia, who had enrolled in the ATM trial, described “You definitely hope for the best outcomes … but we were always reminded that it's still the first stage, phase one of the trial.” Madelaine tried to keep her expectations low but was cautiously optimistic: “I was trying to protect myself … not setting any real expectations. Of course, I wanted him to survive. Of course, I wanted him to be cured if possible … I had that hope, and that was enough to get me through.”

Hope was replenished not so much by clear signs of clinical efficacy but by a lack of signs of poor outcomes. Juanita recalled of the ultrasounds and in utero blood transfusions following the IUHCT: “Each time, we didn't see things digress. We didn't see things get worse from that point, just a little bit better and better each time. So that was really reassuring.” Sasha likewise recalled of the IUERT, “Each consultation, it was basically the same thing. ‘Oh, he looks great. He's the poster child for a fetus.’ So, it was basically like, ‘Oh, he's progressing.’” Sasha trusted feedback from the team but also her mother: “It's really something only the doctors and, I guess, my mother can tell … [as] far as his movement [goes], he's a mover … my mother, she tells me that my brother [who has Hunter's] didn't move that much when he was inside.”

Some parents were still holding out hope for another type of cure at the same time, as well as hope that their choice to participate in the clinical trial may improve knowledge and expand treatment options for themselves and others. Rose said she was holding out hope for a gene therapy, and “All of the stuff that we're doing, it could contribute to other research as well. That could potentially cure the disease for [our daughter] down the line … there's still that small hope that's in the back of my mind. It's like, well, you never know. And even now we know that it didn't work … I wonder if there is some kind of impact. Maybe that's not what the team's testing for. But I wonder if there is some sort of impact that my stem cell has on helping with this disease that may allow her to go longer without a transfusion. If not now, maybe in the future … I know that even if it didn't work with our pregnancy, for this pregnancy it may in the future contribute to a cure. Or this disease that could still be, again, it ties back to the personal aspects, though.” Sasha described of joining the trial: “It's educational and beneficial and gives you a lot of hope. A lot of hope.”

### “I'm my great‐grandmother's advocate”: Parental interests are not limited to the maternal‐fetal dyad

The reasons for participating in phase I clinical trials when there is no expected benefit may be framed in terms of altruism, or what others may stand to benefit from one's participation. The longer‐term question of societal value—the generation of knowledge to improve the health of future fetuses and children—could constitute one of three benefit‐risk analyses (for the fetus, the pregnant woman, and society).^7^ However, it is unclear the extent to which decision‐making for families faced with complicated pregnancies aligns with societal needs beyond their immediate family and social needs.

For Sasha, just as her own mother had “not given up” on Sasha's three‐year‐old brother despite the challenges associated with his untreated condition, Sasha saw the trial as another way to change the narrative about her family's control over loss. The decision partly fulfilled her sense of protecting the next generation. Sasha described herself as wanting “to be the mother that I thought I wanted,” having had a “tough childhood” herself. Her desire for maternal caregiving began with the act of participating in the clinical trial insofar as it preserved respect for the previous family members and their maternal fortitude. Sasha said: “My grandmother said I'm my great‐grandmother's advocate; that my great‐grandmother's looking down on me and so excited and happy that we finally have some type of medicines and research going on about our disease; what we carry.”

Participating in the trial challenged social isolation associated with the disease. As Sasha described: “It's a very lonely thing … to be a carrier of genes that are very rare. You feel like you're the only person in the world going through this…. To even think about being in a clinical trial would be a good thing because you have support.” The feeling of social connection included trust in the clinical research team, who Sasha described as “like a family.” She elaborated, “I didn't feel like just a patient. It felt like a family. Conversations with them outside of speaking on the procedures. I talked to them like they're my best friends.” Sasha described how prior to the clinical trial, “I was not a fan of doctors at all … I wasn't very open with sharing, I felt that our gene was … I want to say a disgrace because I was mad that my brother, my big brother, had passed. I was mad that my uncles had passed.”

Expectant parents were motivated by social worlds beyond themselves. These social worlds included their extended families that have experience with the condition or other families they connected with while considering participation in a clinical trial. Claudia described sharing her story with other families who were considering enrolling in the trial. “I would just let them know, ‘I can only share … what my daughter is now going through.’ But … I just always tell them, ‘You need to decide what's best for you and your family because it's not me.’”

The altruistic desire to give future families a sense of hope, too, was secondary but not absent. Nathan said that their son growing in utero was “the main reason we're doing all of this and I know it's probably going to be more kids … with this same gene … so we just hope this just paves the way for other kids that need the help.” Claudia explained: “I think my whole thing, well, number one was saving my daughter … the second one, why we leaned towards that is because this is a possible cure, and not just for her, it could be a possible cure for others.” Penny echoed this sentiment: “If this doesn't work out, because it's phase one, and we understand there's phase two, phase three, at least they'll come to a better understanding of what does not work and what can we do, how can we change the direction of the research?” Lisa said, “I am so happy to do it because I just I hope that at some point, we can figure it out and they can help and save and cure all these future babies…. And then also, that's less stress for the parent in regards to less medical issues and bills and financial burdens.” Hao pointed out that “we're in a position … to be able to do this, whereas I think [if] we were in a tougher situation even with our families or financially it would've made it harder…. it felt like if we don't do this, the next person that this affects may not be in the same situation, might not have a choice.”

Finally, participants were motivated by the idea that fetal therapy more generally will become socially acceptable because of the hope that it generates as part of a long line of reproductive advancements. As Rose noted, “Once it's successful, it will become widely accepted. Because it does give people hope…. If it's anything like IVF [acceptance of fetal therapy] may take a while. But I think it will become socially accepted. Because I feel like I see more good than harm that it could contribute to the society.” Thus, societal value of these emerging treatments, as viewed by clinical trial participants, could extend to future fetuses who may be eligible for the treatment if it is proven to be safe and effective.

## CONCLUSION

This qualitative study demonstrates that psychosocial reasons are central for families enrolling in maternal‐fetal phase I clinical trials. Clinical trial participation as an attempt to maintain some control and preserve hope appeared to outweigh concern for clinical outcomes of the intervention and clinical trial safety risks. For prospective parents, the alternative of inaction and potential loss were the more relevant risks. There was a temporal focus on getting through the pregnancy and feeling in control during this period, over and above considerations for longer‐term outcomes. Hope and optimism guided decisions to participate, and this was fueled by feelings of social purpose and support within families and the clinical research team. The clinical trials provided a nurturing social experience for families, beyond that which human subjects protections account for. Families were often viewing benefit‐to‐risk ratios from the lens of avoiding loss and changing the narrative moving forward, which calls on clinical research teams to appreciate a well‐rounded appreciation of benefits and risks, and to ensure that families do not harbor unrealistic expectations of medical procedures as they transition to postnatal realities. This paper calls for more research that explores how lived experience intersects with the constructs of hope, optimism, and therapeutic optimism in these fraught clinical‐research contexts.

## ACKNOWLEDGMENTS

The authors thank the families who shared their stories and perspectives. We also thank Tippi C. Mackenzie and C. Alta Charo for their input and helpful conversations. Research in this article was supported by the National Human Genome Research Institute (5R00HG012379‐04) of the National Institutes of Health. The content is solely the authors’ and does not necessarily represent the official views of the National Institutes of Health.
